# Factors influencing women’s entrepreneurial success: A multi-analytical approach

**DOI:** 10.3389/fpsyg.2022.1099760

**Published:** 2023-01-17

**Authors:** Jiaying Feng, Zeeshan Ahmad, Wei Zheng

**Affiliations:** ^1^School of Economics and Management, Harbin University, Harbin, China; ^2^Department of Business Administration, Air University, Islamabad, Pakistan; ^3^Department of Finance, Harbin University, Harbin, China

**Keywords:** entrepreneurial success, female entrepreneurship, influencing factors, structural equation model, artificial neural network, Pakistan

## Abstract

Women entrepreneurs are significant contributors to the economic development of any country and their role becomes more vital in improving the economic condition of developing countries. This highlights the important role of women-owned small and medium enterprises (SMEs) and their entrepreneurial success. Therefore, the current study extends the entrepreneurship literature by examining the effects of factors like personality traits (PT), motivation and commitment (MC), availability of financial resources (AFR), and government support (GS) on entrepreneurial success (ES) of women-owned SMEs. Using a purposive sampling technique data from 255 women-owned SMEs were collected. A multi-analytical approach was employed to analyze the data. The Structural equation modeling (SEM) results indicated that PT, MC, AFR, and GS have a direct effect of ES whereas MC also mediated the link between PT and ES, and the results reveal that in presence of MC the effects of PT on ES become more significant. SEM results revealed that PT and AFR are the most important factors related to entrepreneurial success. On the contrary, ANN analysis revealed that “motivation and commitment” is the most influencing factor. These findings can guide business practitioners and policymakers in the envisioned strategy formulation to encourage women entrepreneurs who can contribute to their country’s sustainable economic growth.

## 1. Introduction

UN developed 17 sustainable development goals (SDGs) with an agenda to achieve these goals till 2030. The fifth SDG is about gender equality, which aims to ensure women’s participation in governance and in public life by creating equal opportunities of economic empowerment for women (United Nations, 2022). UN Women further developed a strategic plan for 2022–25 with an objective to guide and facilitating in achieving the fifth SDG (Canton, 2021). Currently, Small and Medium Enterprises (SMEs) in general and entrepreneurship, in particular, is an indispensable pillar of sustainable economic development, and the role of women in contributing to it has become more significant (Box and Larsson Segerlind, 2018). To stimulate the welfare and prosperity of emerging economies, affirming women entrepreneurs as new engines for sustainable growth is crucial (Ogundana et al., 2021). Existing knowledge on the links among the determinants of corporate success argues that a dearth of clear links exists between individual success factors and the sustainable performance of women-owned SMEs, especially in developing countries. The studies have discussed the importance of women entrepreneurs as a critical “untapped source” of sustainable economic development (Minniti and Naudé, 2010; Sarker and Palit, 2014; Nsengimana et al., 2017; Gupta and Mirchandani, 2018). Nevertheless, researchers indicate the critical role of women’s human capital in the development of the global economy; however, investigation on women’s entrepreneurship is infrequent, (Ahl, 2006; de Bruin et al., 2007; Chatterjee et al., 2018; Mahajan and Bandyopadhyay, 2021). Over the past two decade, many companies founded and led by women have attracted the attention of scholars and practitioners from around the world (Mahajan and Bandyopadhyay, 2021). Therefore, an in-depth analysis of the current study area is required and can make a significant contribution to existing literature to determine the concept systematically and empirically.

Pakistan is an underdeveloped country with a population of 220 million people where women and girls are almost 49% of the overall population (KEMP, 2022). According to a recent report, only 1% of women engage in entrepreneurial activities (Munir, 2022). This indicates that the women have a very limited economic contribution. This limited women’s role in the economic activities of the country make it a subject of debate (Khan et al., 2021). Women in Pakistan are subject to gender discrimination and face inequality, at work place as well as in society, on the basis of gender and very often encounter discrimination and prejudice (Roomi et al., 2018). Governments have initiated several programs to increase the participation of women in the development economic situation of the country, yet the outcomes of those programs have not resulted in an increase in women entrepreneurs. This show the intention of the government in improving women’s participation in economic activities (Batool and Ullah, 2017). Nonetheless, young women still look forward to the government supporting their ventures (Muhammad and Ximei, 2022). Hence government support (GS) is very essential for the entrepreneurial success. GS is very vital for the smooth operations and functional efficiency of the entrepreneur particularly for women, because due to inequality and discrimination they not only require financial support but non-financial support in terms of skill development, relaxation in tax, and access to other resource to achieve sustainable growth and performance (Anwar et al., 2020). Similarly, there are very limited successful women entrepreneurs in Pakistan and it becomes of great importance to study their individual personality traits (PT) that direct them towards success. PT is widely acknowledged contributor towards achieving success (Hachana et al., 2018; Diputra et al., 2021). A large portion of the research conducted in the field of entrepreneurship have considered male entrepreneurs rather than women entrepreneurs. The available literature considering women as entrepreneurs have focused largely on examining the reasons why women become entrepreneurs and start their own enterprise focused on their entrepreneurial behavior (Liu and Tong, 2021; Ojong et al., 2021), or the hurdles women have to encounter while starting their business (Brindley, 2005). Men and women face the problem of starting a business; however, women face anomalies, particularly in rural areas, because of the lack of access to the market, financial support, government support (GS), and other channels. Batool and Ullah (2017) studied the antecedents of successful women entrepreneurship but did not use motivation and commitment as a predictor of entrepreneurial success. Singh et al. (2021) investigated the impact of entrepreneurial orientation and grass-root innovators on entrepreneurial success and recommended conducting future studies to see the impact of personality traits on entrepreneurial success. Moreover, a significant portion of entrepreneurial studies has been done in developed economies (e.g., the United Kingdom, Canada, and the United States of America), and a dearth of studies have been conducted in developing economies (Gupta and Mirchandani, 2018). Similar to other developing countries, Pakistani women face not only unequal opportunities in nutrition, education, and health but also gender inequality and a strict cultural environment for doing business (Rehman and Roomi, 2012). These disparities cause women’s low-level productivity.

Consequently, women empowerment in developing economies in general and in the Pakistani economy is explicitly necessary to provide matching education, health, employment, acquired skills, and opportunities like men’s (Roomi and Parrott, 2008; Yunis et al., 2019). Feudalism, capitalism, and social values can curb the development of women entrepreneurs in their working lives. A woman, as compared to a man, is a systematic subordination decided by patriarchal forces. These factors rigidly restrict career opportunities for women Pakistani entrepreneurs (Roomi and Parrott, 2008). In the context of Pakistan, the insufficient discussion of the vigorous aspects of women’s entrepreneurship motivates us to fill the existing gap. Doing so is essential to identify the link among the micro-level perspectives of the individual-related factors influencing women-owned enterprises in Pakistan. To answer this call, the current research attempts to predict the key determinants that hamper women entrepreneurs from the sustainable functioning of women-owned SMEs in Pakistan.

As the study of Vodă and Florea (2019) concluded that women are less willing to engage in entrepreneurial activity than men because of certain personality traits and challenges. Therefore, this study aims to determine a further understanding of women-owned SMEs and to contribute to the entrepreneurship literature by detecting the factors and challenges that influence the success of women entrepreneurs in Pakistan. To test the research model, this study applied a two-stage analytical approach to measure the most influencing factors contributing to the ES of women-owned enterprises in Pakistan. The integrated methodology is based on structural equation modeling (SEM) and artificial neural network (ANN). The key benefit of integrating both methods is that a new approach can be used to examine the most influential success factors in women’s entrepreneurship. Meanwhile, the pros of one method are employed to offset the cons of the other method (Chong, 2013a). By using the predictive analytical technique, researchers can not only create useful models in practice but also help with explanatory modeling in theoretical construction and testing.

This study is organized as follows. Section 2 discusses the literature associated with the factors affecting women-owned SMEs in Pakistan. Section 3 presents the research methodology. Section 4 provides the survey results. Section 5 presents the discussion and conclusion. Section 6 provides the implications, limitations, and future directions of the study.

## 2. Literature review

### 2.1. The Giessen-Amsterdam model of entrepreneurial success

Frese et al. (2000) proposed an interdisciplinary model for entrepreneurial success. They assumed that entrepreneurial success (ES) relies upon the actions of the entrepreneurs. These actions are derived from the goals and the strategies the entrepreneurs develop as a mission and vision of their venture. This model can be used at both the individual as well as at the enterprise level (subject to the size of the company). At an individual level, when an entrepreneur starts a business (s) he only recruits very few employees and is typically the originator of all the goals and strategies which have a very strong impact on the business. Individuals from the business in pursuit of their own goals. Frese et al. (2000) mentioned that the success of entrepreneurial ventures depends upon the factors like personality, human capital, goals, strategies, and environment. According to this model individuals have different personality dispositions and these unique personality factors help them in achieving their entrepreneurial goals. The environment consists of factors that go beyond the control of the entrepreneur. To deal with the environment these entrepreneurs continuously seek support from different institutions. Motivation is considered a psychological factor and is indicated as the propensity to follow entrepreneurial goals. This model is the best fit for the current research work to explain the phenomenon under observation.

### 2.2. Women entrepreneurship

Entrepreneurship is thought to allow women to achieve their business goals while balancing family and work. As stated by Schumpeter (2020), “women who are innovative, initiative or adopt a business activity are women entrepreneurs.” A women entrepreneur is an individual who starts a self-enterprise with the intent to grab the opportunity, has an incredible vision, a business orientation, with great diligence, and most importantly, a woman who is ready to take higher risks, because she has the spirit of adventure. The economy can grow fast, and poverty can be reduced by treating men and women equally. Literature shows that research on the said topic constitutes approximately 10% of studies on entrepreneurship (Brush and Cooper, 2012; Noor et al., 2022). From the economic and financial perspective, business success refers to the return on assets, sales, earnings, employee growth, and non-pecuniary actions, such as personal growth and achievements (Mcclelland et al., 2005; Gupta and Mirchandani, 2018; Noor et al., 2022). However, Dhaliwal (2000) found that women entrepreneurs quantify success when they perceive economic values, or when they generate revenue and lead their families. That is, once they realize that they are making money and contributing to their family, they become aware of achieving a certain level of success.

Women’s entrepreneurship has drawn extensive attention worldwide (Box and Larsson Segerlind, 2018). Women’s entrepreneurship is also seen as a foundation of entrepreneurial diversity. However, due to the economic and socio-cultural complexities, their talents and potential are often unexploited in developing countries such as Pakistan (Yunis et al., 2019; Noor et al., 2022). This study highlights that there is a dearth of research on the women entrepreneurial environment; however, attempts to understand it in isolation recommend only limited insight, regardless of background.

Moreover, several factors are associated with mobilizing, such as women, to become successful entrepreneurs. However, success is an intrinsic situation, referring to increasing financial yields, self-governing, controlling one’s own future, being your own boss, own revenue, and capital gains (Paige and Littrell, 2002; Li C. et al., 2020). ES refers to entrepreneur outputs and achievements in business. Occasionally, achievements can measure the number of employees, the amount of sales volume, and the increase in revenue. The results of the efforts wielded by entrepreneurs are influenced by several factors (Abasilim, 2015). Similar conditions are also applied to women entrepreneurship. Such conditions improve the motivation and performance of women-owned businesses. Women entrepreneurs generally concentrate on power in controlling their own fate, strengthening relationships at the customers’ level, to develop something valuable (Gautam, 2016).

The participation of women in business activities, specifically as entrepreneurs showing encouraging improvements in the growth of women entrepreneurship around the globe, has been contributing to national development since the last decade (Hassan et al., 2014). In Pakistan, women find participating in business activities difficult due to the dearth of socio-cultural and economic freedom. Women entrepreneurs may have fewer opportunities than men because of innate gender discrimination, which is considered a general issue in all developing economies (Rehman and Roomi, 2012). Moreover, lack of availability of financial resources (AFR), training, personality traits (PT), and government and family support are the other significant factors, which confine women’s spatial mobility and discourage them from becoming entrepreneurs in developing countries, such as Pakistan, (Mcclelland et al., 2005; Roomi and Parrott, 2008; Narayanasamy et al., 2011; Mustapha and Subramaniam, 2016). ES is influenced by formal and informal support from others; formal support is provided in the form of financial, technical, and industrial liaisons, whereas informal support refers to personal-and community-based networks (Carrier et al., 2004; Makhbul and Mohamad Hasun, 2010). The current study intends to measure the hypothetical association among urging factors contributing to the achievements of women entrepreneurs in Pakistan.

### 2.3. Personality traits

Entrepreneurial personality traits have gained the attention of the scholars during past few decades. PT are the specific characteristics of an individual including their emotions, way of thinking, and behaving (McShane and Von Glinow, 2010). According to empirical findings of some researchers’ personality traits are an essential part of the behavioral dispositions of entrepreneurs, whereas many share their opinion that there is no connection between personality dispositions and the behavior of an entrepreneur. According to Franco and Prata (2019), entrepreneurs require specific dispositions in order to pursue their goals. Sarwoko and Nurfarida (2021) mentioned that internal locus of control, motivation to achieve goals, moderate level of risk-taking propensity, creativity, capacity to tolerate stress, along with higher levels of self-efficacy are key dispositions required by an entrepreneur (Kusumawijaya and Astuti, 2021). In a knowledge-based economy creativity and innovation have been highlighted as significant aspects of personality traits and have been termed as the psychological intelligence element of human beings and knowledge is regarded as the considered as the critical element to develop this personality trait (Zhou et al., 2021).

### 2.4. Motivation and commitment

Push and pull factors are the key driving forces and motivations for women to become an entrepreneur. Push factors are the negative circumstances or situations that make woman start her entrepreneurial venture. Factors such as unemployment, too much idle time at a previous job, too much pressure and expectation from the previous employer, family circumstances expecting the women to take care of the family, flexible working hour requirement, (Leyton-Román et al., 2021), On the contrary, pull factors are the positive factors to make a choice about pursuing the entrepreneurial venture. These are the innate desires of women to become entrepreneurs. These are caused by the concept of independence, being your own boss, autonomy in decision-making, and a sense of self-achievement, and desires for being creative, have power and making handsome earnings (Troise and Tani, 2020). Women in most developing countries start entrepreneurial ventures due to a desire to improve their socioeconomic status, fulfilling their sense of achievement or improve their social status (Leyton-Román et al., 2021). Sometimes these startups are not their priority rather a result of the poor economic situation, loss of bread earners in the family, or a result of social discrimination.

### 2.5. Availability of financial resources

Resources available to start the entrepreneurial adventure are very critical and they become significantly important for women entrepreneurs (Salehi et al., 2019). Access to financial resources are of great importance. Easy access to banks and financial institutions make it easier to avail the credit facilities. Keeping this in view several national and international organizations are emphasizing the availability of these facilities to promote women’s entrepreneurship and economic development (Adomako and Ahsan, 2022). Under instable economic situations AFR enables the entrepreneurs to develop strategic orientation to cope up with economic crisis that is why many commercial banks are ensuring that they also play their role to promote creative, and economically viable start-ups initiated by women (Beliaeva et al., 2020). Eggers (2020) mentioned AFR as the master of disasters because AFR can shield the SMEs from the jeopardized market, crisis in the economy, or any other external shocks. AFR also facilitate in developing intellectual, customer and social capital that are necessary to work efficiently by the entrepreneurs (Li G. et al., 2020).

### 2.6. Government support

Women entrepreneurs need support not only from their families but also from other institutions and government support is very vital for the success of women entrepreneurs (Hu et al., 2022). GS help new startup to reduce the fear of closing the business due to lack of experience, helps in examining the strengths, weaknesses, external threats and opportunities arising from internal as well as external environment. Park et al. (2020) mentioned that GS also act as a guide with an objective to widen the SMEs benefits to the wider community. Most importantly GS facilitate in developing the differentiation strategies which enable SMEs to become more sustainable. With all the governmental interventions GS can becomes beneficial to eliminate the market bias towards SMEs. Keeping this in view governments are establishing women’s chambers of commerce, ensuring that women entrepreneurs get all the facilities and support required to make their start-ups a success (Rehman et al., 2022). It provides support for every women entrepreneur to ensure that their start-up smoothly becomes operational and starts making an economic contribution. Still, the other government departments need to be synchronized so that they also extend their support and facilitate these women entrepreneurs in their start-up (Obschonka et al., 2019).

## 3. Research framework and hypotheses development

**Figure 1:**
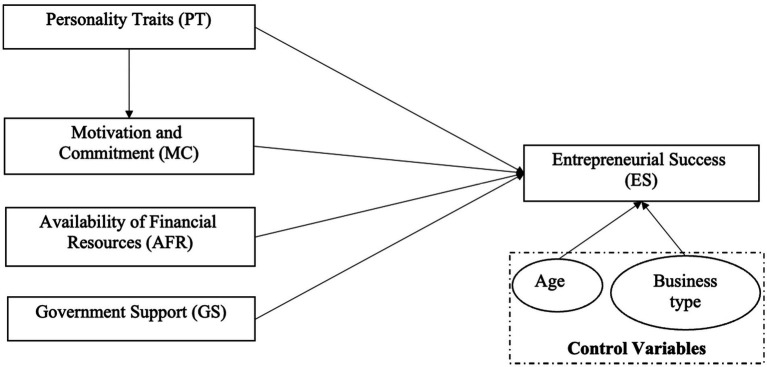
Theoretical framework.

Grounded in the literature review, this research offers a framework to analyze the relationship between influencing factors. Figure 1 represents the current study framework.

### 3.1. Entrepreneurial success

Successful entrepreneurs play an important role in societal development because they help create jobs and promote economic growth (Epezagne Assamala et al., 2022). However, a generally accepted definition of success, specifically for the new enterprise environment, does not exist because no mutual agreement is found among various authors (Oyeku et al., 2014; Chatterjee et al., 2018; Qader et al., 2022). ES simply refers to tangible elements, such as organizational growth, capital formation, sustainability, revenue turnover, and organizational sustainability (Dafna, 2008; Makhbul and Mohamad Hasun, 2010). Harada (2003) argued that certain entrepreneurs retain their position and business despite facing loss and other difficulties due to their high-level determination in PT. From the perspective of women entrepreneurs, success means perceived economic value and creating a sophisticated income level, which helps lead their family (Hasan and Almubarak, 2016; Gupta and Mirchandani, 2018; Yunis et al., 2019; Li C. et al., 2020). Initially, it belongs to this sector of women’s life instead of defining and achieving other business goals. Success is a key term used in business management that can be measured as performance, income level, and profitability. The characteristics of creativity and excellent interpersonal, psychological, and technical skills contribute to the success of entrepreneurs (Li et al., 2018; Tshiaba et al., 2021). Therefore, ES is given two definitions in this study context. First, ES is referred to as the women state of perception or stratification regarding the achievement of goals set to become the spearhead of the house by running a business. Second, ES should measure the organizational growth and expansion of women-owned businesses.

### 3.2. Relationship between PT, MC, and ES

Literature shows that PT can affect ES in terms of motivation, internal locus of control, moderate risk propensity, productivity, stress tolerance, innovativeness, and generalized self-efficacy (Brandstätter, 2011; Gupta and Mirchandani, 2018). Personal factors also enhance education, motivation, and commitment levels. Entrepreneurs’ education and experience can not only improve the understanding of business activities but also assist in polishing their personality, which helps entrepreneurs become successful (Brandstätter, 2011; Gupta and Mirchandani, 2018; Vodă and Florea, 2019). PT is also associated with women’s decision to become successful entrepreneurs (Naser et al., 2009). Entrepreneurial education was found to develop entrepreneurial personality traits in college students and thus results more entrepreneurial intention among these students, (Zhou et al., 2021). Moreover, Zhou et al. (2021) revealed that the knowledge is the professional base for developing the entrepreneurial personality in individuals that motivate them towards start ups.

Past literature has also indicated that individual personality traits are the main reason that motivated them to become an entrepreneur (Chen and Lai, 2020). Entrepreneurs have unique personality dispositions that develop the motivation and commitment toward entrepreneurial start-ups (Ismail, 2022). Personality-job fit theory supports the concept that a match between the personality traits of an individual and the task they perform and this person-job fit defines the success of the individual in the career they select (MacKenzie, 1995). Social networks also play a vital role in developing psychological motivation and commitment among individuals to ensure that they succeed in their business initiatives (Jiatong et al., 2021). Based on these arguments, it is concluded that certain personality traits can also lead women entrepreneurs toward entrepreneurial success. In addition, motivation and commitment among women entrepreneurs keep them focused on achieving success. Therefore, this study hypothesizes the following:

*H1: PT has a significant effect on the success of women's entrepreneurship.*

*H2: PT has a significant effect on the MC of women's entrepreneurship.*

### 3.3. Relationship between MC and ES

Literature shows that family support is a significant element in the creation of entrepreneurship interest. Family support includes the support from the husband, guardians, and other family members for the successful initiation and growth of a business (Winn, 2004; de Bruin et al., 2007; Jennings and Mcdougald, 2007). Family support can help motivate women, especially in developing countries, to become entrepreneurs. Meanwhile, push and pull factors are associated with women’s motivation and commitment (MC) to become entrepreneurs. Push factors include redundancy, previous job frustration, unemployment, work schedule flexibility, and the requirement to earn a rational living standard. Pull factors comprise independence, being one’s boss, autonomy, self-fulfillment, using creative skills, self-achievement, entrepreneurial drive, wealth desire, doing enjoyable work, power, and social status (Uddin and Kanti, 2013; Gupta and Mirchandani, 2018; Zain et al., 2022). The above presented arguments help in building the assumption that highly motivated and committed women have more orientation to achieve success in their entrepreneurial ventures. Therefore, this study aims to identify the association between women’s enthusiasm to become successful entrepreneurs and hypothesize the following

*H3: MC has a significant effect on the success of women's entrepreneurship.*

### 3.4. Relationship between AFR and ES

Financial support is another essential factor that may influence women’s decision to become entrepreneurs. AFR is the primary factor in the creation, growth, and survival of any start-up business (de Bruin et al., 2007; Gupta and Mirchandani, 2018). SMEs need additional financial resources for their expansion to meet the cost of market expansion, thereby running research and development activities (Naser et al., 2009). Women operating these SMEs can generate a massive amount of capital by themselves; capital is available through debt financing from the banking sector or initial public offerings (Masuo et al., 2001; Jennings and Mcdougald, 2007). Meanwhile, the AFR can help women entrepreneurs to access the market, which is also a key factor to success (Narayanasamy et al., 2011). ARF is found to develop resilience in SMEs to counter environmental challenges by enabling them to perceive the uncertainties and develop capabilities to successfully complete their goals (Gayed and El Ebrashi, 2022). The AFR motivates individuals to invest more in their ventures, and be more engaged in business activities and this helps them perform better (Adomako and Ahsan, 2022). Furthermore, Anwar and Li (2021) shared that the availability of financial resources helps SMEs in improving their governance system to achieve sustainable performance. The above arguments propose that the availability of financial resources for women entrepreneurs is a vital factor that can eliminate several reasons for failure in their ventures and direct them toward success. Therefore, the following is proposed.

*H4: AFR has a significant effect on the success of women's entrepreneurship.*

### 3.5. Relationship between GS and ES

The environment for businesswomen, particularly in developing countries, may prevent them from becoming successful entrepreneurs. Gender inequality exists in access to the market because women-owned SMEs can obtain the same benefits as their male equivalents (e.g., access to information, business registration, start-up capital, and marketing opportunity) in developing countries (Entrepreneurship, 2004; Welter, 2004; Gupta and Mirchandani, 2018). Meanwhile, the unavailability of GS schemes and the lack of government interest in women’s entrepreneurship have created obstacles and increased challenges for women’s long-term entrepreneurship sustainability (Naser et al., 2009; Yunis et al., 2019). Nevertheless, government-women associations and non-government organizations (NGOs) can play critical roles in mitigating gender inequality by creating and implementing regulations for the success of businesswomen in addition to family support (Uddin and Kanti, 2013; Sarker and Palit, 2014). Pulka et al. (2021) argued that GS provides all the required support to SMEs that can develop skills and capabilities in the entrepreneurs to achieve better performance goals and make their venture successful. Ishtiaq et al. (2020) highlighted GS as an essential external factor that can contribute to SMEs’ success. GS support eliminates the barriers to implementing the technology in their organizations successfully and leads the business to improve its financial performance (Jayeola et al., 2022). Moreover, Anwar and Li (2021) shared that financial or non-financial support helps improve the governance system of SMEs and facilitate them in becoming sustainable. These factors can assist in improving women’s participation and success rate in the business field, both of which are ultimately required for the sustainable development of a country. Therefore, this study hypothesizes the following.

*H5: GS has a significant effect on the success of women's entrepreneurship.*

### 3.6. Mediating role of MS

Cheng (2019) conducted an investigation to examine the effects of personality traits of entrepreneurs and their motivation and commitment towards achieving entrepreneurial success and concluded that personality traits are the key dispositions that develop the motivation among individuals to pursue their entrepreneurial start-ups. These personality dispositions help entrepreneurs to perform better and stay committed to improving their performance (Chen and Lai, 2020). The personality traits of an entrepreneur develop the willingness to be creative and improve their self-efficacy (Liu et al., 2019). Similarly, emotional competence was found to keep motivated the university students towards their social entrepreneurial projects (Chien-Chi et al., 2020). Moreover, creative university students were found more motivated and committed and displayed higher intentions to pursue entrepreneurial projects and direct them toward success (Shi et al., 2020). The discussion provides an observation that the MS plays the role of a mediator and therefore the current hypothesis is:

*H6: MC mediates the relationship between PT and ES.*

## 4. Methodology

### 4.1. Sampling and data collection

Survey methodology is the most viable technique when it comes to collecting data in a shorter period and from a large group of people when they are scattered. It is also the most cost-efficient technique in the data collection (Granello and Wheaton, 2004). Therefore, a survey methodology was used and a questionnaire was designed for collecting the data from the women who run SMEs in Pakistan. The survey questionnaire was designed to collect the responses against multiple questions on a Likert scale of seven points (Table 1). This scale ranged from “1 = Strongly Disagree” to “7 = Strongly Agree.” The previous researcher provides sufficient evidence in support of using the seven-point scale to collect data in comparison to other techniques of data collection (Preston and Colman, 2000; Pasek and Krosnick, 2010). Data collection process was assisted by using the Computer-Assisted Web Interview (CAWI) technique, in this technique the respondents respond to the questionnaire using the computer with the assistance or guidance provided to them at that moment. Women-owned SMEs were selected from the database of the small and medium enterprises development authority (SMEDA), Pakistan. Purposive sampling was used to select the firms where women were actively operating in Punjab province. They were contacted to participate in the study *via* phone or email obtained from their profile information available in the SMEDA database. After getting their consent to participate, a link to the survey was sent to them. Questionnaires were sent to 270 respondents and only 255 were valid responses that were considered for the analysis. The participants participated on a volunteer basis, they also participated by agreeing to provide data without any monetary or non-monetary incentive. The participants were well informed about the objectives of the study before their consent, motivating them to take part in the study survey. All the elements of the selected constructs were adapted from preceding literature to confirm content validity.

**Table 1:** Survey questionnaire.

Sr.	Items	Source
Personality traits (PT)		
1	Knowledge about the business affect my involvement in running a self-enterprise	Naser et al. (2009) and Gupta and Mirchandani (2018)
2	My interest and hobbies affect my participation in running a self-enterprise	
3	Personal skills and experience are required for the successful growth of self-enterprise.	
4	Profit motive affect my involvement in developing self-enterprise	
5	I believe that determination and innovativeness are essential for running a success self-enterprise.	
6	I believe that stress tolerance is also essential for entrepreneurial success.	
Motivation and commitment (MC)		
7	Doing enjoyable work with a flexible schedule motivates me to start self-enterprise.	(Naser et al., 2009), self-constructed
8	Responsibility for households in residence affects my involvement in developing self-enterprise.	
9	Father’s occupation affects my involvement in running a self-enterprise.	
10	I involved in developing self-enterprise because I want to earn money and social status.	
Availability of financial resources (AFR)		
11	Start-up capital is vital for the successful expansion of self-enterprise.	Naser et al. (2009)
12	Access to credit from government/non-government organizations is important in developing successful self-enterprise.	
13	Access to credit from banks is important in the development of a successful self-enterprise.	
Government support (GS)		
14	Government policies are important to my involvement in developing a self-enterprise and become a successful entrepreneur.	Naser et al. (2009)
15	Government and non-government support are important to my involvement in developing self-enterprise.	
16	Participation in women association is essential to increase the success rate of women entrepreneurship.	
Entrepreneurial success (ES)		
17	The sales of my enterprise are potentially grow over time.	(Gupta and Mirchandani, 2018), self-constructed
18	My enterprise is getting market share.	
19	My enterprise is earning profit gradually since I started.	
20	I am confident about the survival of my business in the future.	
21	I am planning to extend my business activities.	

### 4.2. Study context

This investigation aims to determine the factors affecting women’s ES in Pakistan using a multi-analytic approach; therefore, the proposed hypotheses were tested by using primary data collected from Pakistan. The research data was collected from the South Punjab region of Punjab province, which is administratively divided into two—Bahawalpur and Multan. The province has a mix of population distributed in the diverse urban areas and rural deserts, such as Cholistan and Thal, with low population density. The study collected primary data through a questionnaire from administrative divisions, such as districts, tehsils, and union councils, to ensure representations of all segments, communities, geographical areas, urban–rural areas, and scattered and congested parts of the study population. This helped in identifying and collecting the research data from the women entrepreneurs of the region and contribute towards the objectives of the study. This research was performed from August-2022 to November-2022.

### 4.3. Demographics of respondents

The demographics of the respondents were evaluated by applying frequency and descriptive analysis methods. The results in Table 2 show that the total valid number is 255, representing the sample size of this study, out of which a large proportion of the individuals responding to the questionnaire belonged to the age group of 25–34 (56.5%), whereas 20.4% are 18–24 years old. According to the results, most respondents are single. Meanwhile, 28.2% of the respondents attained secondary education, and the same percentage obtained bachelor’s degrees. Moreover, above 70% of the respondents are engaged in trading and other types of businesses, but only 11% are engaged in manufacturing. Most respondents have less than 6 years of work experience, whereas 30.2% have more than 10 years of work experience. The results in Table 2 suggest that young, single, and educated women with good work experience in trading and other types of business activities are the respondents of this study.

**Table 2:** Demographics of respondents.

Category		Frequency	Percent
Age	18–24 years	52	20.4
	25–34	144	56.5
	35–44	31	12.2
	45 years and above	28	11
	Total	255	100
Marital status	Single	183	71.8
	Married	72	28.2
	Total	255	100
Education	Primary	15	5.9
	Secondary	80	31.4
	Higher secondary	72	28.2
	Bachelor	72	28.2
	Master	16	6.3
	Total	255	100
Business type	Service-oriented business	40	15.7
	Manufacturing	28	11
	Trading	66	25.9
	Other	121	47.5
	Total	255	100
Experience	1–3 years	75	29.4
	4–6 years	79	31
	7–10 years	24	9.4
	10+ years	77	30.2
	Total	255	100

## 5. Findings

### 5.1. Data analysis methods

Before the execution and screening of data for applying SEM, exploratory factor analysis was first performed employing SPSS 25. As per the recommendations of Anderson and Gerbing (1988) the data was evaluated using a two-step process. In first step, the scrutiny of construct validity was performed by estimating the reliability values, estimating the measurement model, and observing the values for a good fit model to see the convergent validity and discriminant validity of the analyzed data. In the second step employing the structural equation model (SEM) the proposed links were tested to verify the relationships of the proposed framework. Considering the exploratory nature of this research, we applied SEM to perform analysis using AMOS 23 (Fornell and Larcker, 1981; Rajalahti and Kvalheim, 2011). Meanwhile, applying the ANN model the most influential factor predicting entrepreneurial success among women entrepreneurs was also estimated as it could not be estimated using traditional methods like multiple regression analysis, logistic regression, and SEM (Chong, 2013a; Sharma et al., 2015). Furthermore, ANN is capable of calculating nonlinear relationships as it estimates the performance of the task similar to the workings of the human brain therefore is considered one of the most influential statistical learning models to estimate human brain-like preferences.

### 5.2. Measurement model

The measurement model was applied to confirm the reliability, convergent, and discriminant validity of the study. The value of Cronbach’s alpha in Table 3 describes that the results of the reliability analysis for PT is 0.932, MC is 0.890, GS is 0.871, AFR is 0.884, and ES is 0.940. This value is higher than the prescribed value of 0.7. At the same time, the significance criterion of the factor estimation coefficient was used to test the convergent validity of the related structural factors. Therefore, principal component analysis using Varimax rotation was applied to explain the key factors that help in the success of women entrepreneurs in SMEs in Pakistan. All the loading values are above 0.7 and range from 0.709 to 0.839 within their respective factors. To estimate the sampling suitability, Kiser-Meyer-Olkin (KMO) test and Bartlett’s test for sphericity were also applied (Bartlett, 1954; Kaiser, 1970). The KMO value is 0.929, and Bartlett’s test for sphericity is significant at 0.001 level.

**Table 3:** Results of factor loadings, convergent validity, reliability, and descriptive statistics.

SR	Variables	Items	Loadings	Cronbach’s alpha	CR	AVE
1	Personality trait	PT1	0.817	0.932	0.932	0.696
		PT2	0.789			
		PT3	0.818			
		PT4	0.709			
		PT5	0.836			
2	Motivation and commitment	MC1	0.778	0.89	0.89	0.67
		MC2	0.808			
		MC3	0.758			
		MC4	0.812			
3	Government support	GS1	0.816	0.871	0.871	0.693
		GS2	0.796			
		GS3	0.775			
4	Availability of resources	AFR1	0.839	0.884	0.885	0.719
		AFR2	0.792			
		AFR3	0.779			
5	Entrepreneurial success	ES1	0.833	0.94	0.941	0.762
		ES2	0.801			
		ES3	0.814			
		ES4	0.716			
		ES5	0.714			

Later, using the confirmatory factor analysis (CFA) method, the composite reliability (CR), item loadings, and the average variance extracted (AVE) for latent constructs were estimated. The results revealed that the estimated values are greater than 0.5 the set threshold; the outcome values estimated for the measurement model are shown in Table 3 (Fornell and Larcker, 1981; Rajalahti and Kvalheim, 2011). In conclusion, the relevant results estimated using CFA suggest that all items or the latent constructs are consistent.

The discriminant validity was checked by estimating the inter-construct correlation and the square root of the AVE, and the estimated results are shown in Table 4. Fornell and Larcker (1981) have recommended that while estimating the results of construct correlation it is important to note that the for construct it corresponding values of average variance shared is higher than the values against other constructs., The results in Table 4 show that the square root of AVE for reflective constructs is consistently higher than the inter-construct correlations, significantly describing the discriminant validity among the constructs. The values of model fit indices during CFA analysis are also under the accepted range (Rajalahti and Kvalheim, 2011); Chi-square/df is 1.980, CFI is 0.959, SRMR is 0.039, Root Mean Square of Error (RMSEA) is 0.062, PClose is 0.019, and TLI is 0.952. Thus, the CFA results indicate the goodness of fit of a statistical model. Moreover, no issue of discriminant validity is found, and the data can be used for SEM analysis.

**Table 4:** Inter-construct correlations and validity concerns.

	MSV	MaxR(H)	PT	ES	MC	AFR	GS
PT	0.405	0.938	**0.834**				
ES	0.415	0.949	0.636***	**0.873**			
MC	0.398	0.893	0.533***	0.631***	**0.819**		
AFR	0.415	0.888	0.519***	0.644***	0.546***	**0.848**	
GS	0.383	0.872	0.535***	0.619***	0.588***	0.597***	**0.832**

Significant at: Inclined lines rendered in Boldface show the Square Root of the AVE. Note: PT = personality traits; ES = entrepreneurial success; MC = motivation and commitment; AFR = availability of financial resources; GS = government support.

*** *p* < 0.001 (Significance).

### 5.3. Structural model

**Figure 2:**
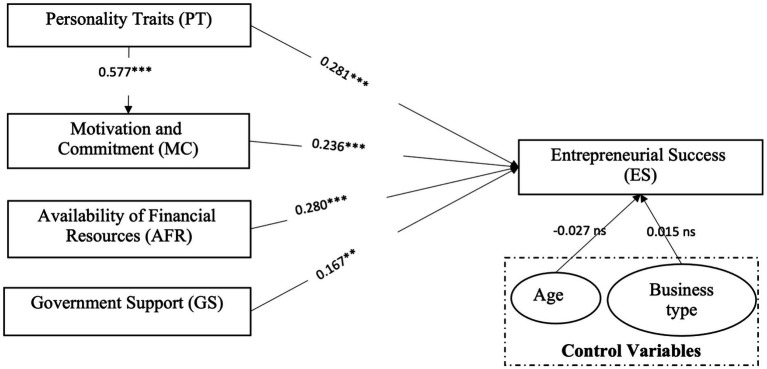
Structural model.

SEM was applied to validate and measure the proposed hypotheses. Figure 2 displays the results the after performing the analysis of SEM. Overall, values were measured in a standardized form, and the value of R-square is 0.66, indicating that the proposed variables result in an overall 66% variation in the dependent variable. Age and type of business were considered control variables in this study, and both do not affect ES. We also observed the goodness of fit of a statistical model, and Figure 2 shows that the value of Chi-square/df is 1.726, CFI is 0.991, SRMR is 0.053, PClose is 0.398, and TLI is 0.979, all of which meet the recommended values of good model fitness (Fornell and Larcker, 1981; Rajalahti and Kvalheim, 2011). Therefore, we can presume that this model is a good fit and may not influence the results of the path coefficient.

The results of the path coefficient are significant and describe the positive effects of the selected constructs on the ES of women-owned SMEs. The standardized value of the path coefficient for PT is 0.281, which is significant at *p* < 0.001. Therefore, PT is an essential contributor to the ES of women-owned SMEs in Pakistan. The standardized value of the path coefficient for PT to MC is 0.577, which is significant at *p* < 0.001. Therefore, PT is an essential contributor to the MC of women-owned SMEs in Pakistan. Meanwhile, the path coefficient of MC is 0.236, which is significant at *p* < 0.001. Therefore, MC also positively contributes to ES. Furthermore, AFR is also an important predictor, which positively affects the ES of women-owned SMEs (*b* = 0.280, *p* < 0.001). Finally, the standardized value of the path coefficient for GS is 0.17, which is significant at *p* < 0.01.

The results clearly indicate that the goof fit for the overall path model, and all the constructs significantly and positively contribute to ES. Business type and age do not have any significant impact on the ES of women-owned SMEs. Grounded on the SEM results, we can accept the proposed hypotheses in Table 5. We now conclude that “PT” and “AFR” are the most influencing factors affecting the ES of women-owned SMEs, followed by MC and GS.

**Table 5:** Hypotheses results.

	Estimate	S.E.	C.R.	*p*	Remarks
ES < --- PT	0.281	0.054	5.205	***	H1 = Accepted
MC < --- PT	0.577	0.051	11.249	***	H2 = Accepted
ES < --- MC	0.236	0.045	5.241	***	H3 = Accepted
ES < --- AFR	0.28	0.051	5.454	***	H4 = Accepted
ES < --- GS	0.167	0.052	3.193	**	H5 = Accepted
ES < --- Age	−0.027	0.043	−0.638	ns	No effect
ES < --- Business type	0.015	0.033	0.443	ns	No effect

ns = not significant. PT = personality traits; ES = entrepreneurial success; MC = motivation and commitment; AFR = availability of financial resources; GS = government support.

** *p* < 0.01 (Significance).

*** *p* < 0.001 (Significance).

### 5.4. Mediation analysis

Furthermore, according to the results shown in Table 6, MC mediates in the relationship between PT and ES as shown in Table 6. Hence, H6 is accepted.

**Table 6:** Mediation Analysis.

	Hypothesis	Direct effect	Indirect effect	Results
H6	PT → MC → ES	0.427***	0.246***	Supported

PT = Personality Traits; ES = entrepreneurial success; MC = motivation and commitment.

*** *p* = <0.001.

### 5.5. Artificial neural network model

Tadeusiewicz (1995) described ANN as a group of interlinked nodes, which are inspired by the simplest mechanism of neurons of a brain. In ANN each node is a representation of and neuron and each link connecting these neurons is represented by the arrows which create the link between these nodes, and these arrows for the output from one neuron to another neuron and becomes the input for the other neuron. ANN model is regarded as more robust model which provide high accuracy in predicting the preferences which is far more reliable than the output obtained from the conventional regression estimation approached like multiple regression analysis and SEM (Chong, 2013a). Moreover, ANN model work on the pattern of a human brain which makes it an efficient statistical learning model. ANN model fundamentally works using the interlinked processing units. These interlinked units are known as the neurons. ANN model is comprised of three layers, which are the input layer, the hidden layer, and the output layer. The independent variables are the predictors and work as the input layer in ANN model, whereas output layer is formed using the dependent variable (s). Further, the hidden layers are of two types. One hidden layer represents the continuous function of the ANN model, whereas the two hidden layers in ANN model represent the discontinuous model. Meanwhile, concerning previous studies, particularly related to technology adoption and acceptance, have used a single hidden layer while predicting the preferential estimates using ANN model (Chong, 2013a,b; Sim et al., 2014; Sharma et al., 2016, 2017; Liébana-Cabanillas et al., 2017; Tolani et al., 2019). In an ANN model, the synaptic weight is the weight assigned to each layer which usually is not zero, whereas the hyperbolic tangent in any layer or node is represented by the linear or non-linear activation function in ANN model (Chong, 2013b; Sharma et al., 2016; Liébana-Cabanillas et al., 2017). ANN is popular method used in the area of information system research due to the fact that it is convenient to use, provides robust, reliable computing power, and provide accurate prediction of adoption factors.

Current study utilizes the strengths of ANN model to predict the most significant predictor of measuring ES, which to best of our knowledge, has not been covered till now. Using the SPSS v25, current study used the Back-Propagation Multilayer Perceptron type of ANN model to predict the most influential predictor of ES. After estimating the results of SEM, PT, MS, AFR, and GS are used as the input layer while using ANN model, whereas the ES (the dependent variable) formed the output layer in ANN model. The past literature did not specify any specific number of hidden layers of neurons while prediction estimation using ANN model. Consequently, as per the suggestions by Sharma et al. (2017) 1–10 different layers of hidden neurons were used as hyperbolic tangent activate functions to estimate the identity of the hidden layers and predicting the output layer (Sharma et al., 2016, 2017). The results on the seventh node in a hidden layer are considered complex and accurate to predict the importance of input variables because it shows the higher value in training and testing of ANN model (Table 7).

**Table 7:** Validation results of ANN model (RMSE values).

Network configures	Training	Testing
ANN1	0.405	0.443
ANN2	0.418	0.392
ANN3	0.383	0.439
ANN4	0.416	0.339
ANN5	0.417	0.404
ANN6	0.432	0.418
ANN7	0.448	0.45
ANN8	0.407	0.43
ANN9	0.386	0.406
ANN10	0.39	0.37
Average	0.41	0.409
Standard deviation	0.02	0.035

### 5.6. Validation of the artificial neural network model

In order to avoid the data overfitting, ANN analysis were conducted using a 10-fold cross validation, network training data were taken as 70% and the 30% represented the testing data to predict the accuracy of the training network data (Chong, 2013b; Sharma et al., 2017). Predictive accuracy was checked by estimated the root mean square errors (RMSE) as an indicator for prediction accuracy for the network and testing data for all 10 neural networks while predicting the ANN model analysis. Afterwards, the standard deviation (SD) value and the average for both these data sets were also measured and the results are shown in Table 7. Table 7 clearly shows that the average for the training data set was 0.410 and for the testing dataset was 0.409. Similarly, the SD values for the training dataset was 0.020 and for the testing dataset SD value was 0.035. The RMSE values estimated through ANN were found to be small, revealing that the ANN model has predicted the accurate and reliable values.

### 5.7. Results of sensitivity analysis

The sensitivity analysis is a test which facilitate in estimating the importance of every predicting variable and show the extent to which the values estimated through network model bring change to the independent variable (Chong, 2013b). The results presented in Table 8 clearly signifies that “MC” is the most influential factor predicting “ES.” “PT” turns to be the second most important factor that plays important role in predicting the “ES” among the women entrepreneurs in Pakistan, followed by “AFR” and “GS.” Moreover, Table 8 also show the arrangement of important predictors of ES on the basis of normalized importance score from very important to low importance order.

**Table 8:** Independent variable importance.

Variables	Importance	Normalized importance
Motivation and Commitment	0.368	100.00%
Personality Traits	0.322	87.30%
Availability of Financial Resources	0.182	49.30%
Government Support	0.128	34.80%

## 6. Discussion

This research aims to identify the crucial success factors of women entrepreneurs in SMEs in Pakistan. Therefore, on the basis of the literature review, particularly related to developing countries, this study proposes a research model that has been tested empirically using responses from Punjab province, Pakistan. All the responses were collected using an online survey method with the help of a structured questionnaire, which was adapted from the literature for this hypothetical study. To test the proposed hypotheses, we employed SEM using AMOS, which verified the influencing impacts of the selected factors that can potentially contribute to the success of women entrepreneurs in Pakistan.

The results of first hypothesis indicate that “PT” is significantly affecting the ES. These results highlight that the individuals having the knowledge, interest, personal skills and expertise, firm believe to consider innovativeness as the key driver for operating self-enterprise and developing a tolerance for stress and entrepreneurial challenges facilitate such individuals to become successful entrepreneurs. These findings are aligned with past research which also concluded that locus of control and self-achievements are the significant personality dispositions that help create the entrepreneurial ventures (Vodă and Florea, 2019). The results of the second direct hypothesis emphasized that when entrepreneurs are motivated and committed to self-enterprise they take responsibility of developing their venture, enjoy working in flexible hours, and take more interest in self-enterprise to make wealth and improve social status. Prior research revealed that MCs are the basic requirement for autonomous enterprise and direct the entrepreneurs to achieve higher performance (Leyton-Román et al., 2021). Meanwhile, “MC” is also evidenced as a significant factor, which is based on the family support that can boost the success of women entrepreneurs in running SMEs in Pakistan. This result not only enhances women’s confidence to become entrepreneurs but also increase the success of their business activities, which can ultimately contribute to the national development of Pakistan. Next, the results highlighted that “AFR” is highly related to the ES of women-owned SMEs. Such results suggest that PTs of women entrepreneurs can lead them to ES if they have sufficient financial resources. The findings of this investigation are generally supported by various prior studies (Uddin and Kanti, 2013; Sarker and Palit, 2014; Mustapha and Subramaniam, 2016; Gupta and Mirchandani, 2018). Moreover, respondents gave an excellent weight to “GS,” which is based on the role of government to assist women in achieving their entrepreneurial goals. These results are also consistent with those of previous studies (Chowdhury, 2012; Gupta and Mirchandani, 2018), concluding that GS is believed to be one of the leading influential aspects in the growth of any country’s prosperous entrepreneurship, specifically in developing countries. The results of the mediation analysis highlighted that MC positively mediates in the link between PT and ES. These findings are similar to past study which highlighted that specific PT are pre-requisites to keep MC higher among the entrepreneurs to achieve success in their ventures (Chen and Lai, 2020).

In extension to the SEM analysis, the ANN model was applied for the first time in this stream of research. Doing so helps understand the most influential factors predicting the success of women entrepreneurs in Pakistan. Unlike the results obtained from the SEM analysis, the results from the ANN analysis described that “MC” is the most influencing predictor of ES among women in Pakistan. Meanwhile, “PT” is the second most influencing factor, followed by “AFR” and “GS.” However, “MC” is the third most important factor based on the SEM results. This result is possible because the ANN model detected the nonlinear relationship among the deciding factors, rather than the structural model. Overall results support that a committed woman with strong entrepreneurial motivation, personality, and AFR is prepared to accept the challenges of women-owned SME operations, which contributes to the sustainable development of her country.

## 7. Conclusion, implications, and future directions

Women entrepreneurs are becoming a significant contributor to the development of the economic in any country and their role becomes more vital in improving the economic condition of developing countries. Therefore, this study investigated the associations between PT, MC, AFR, GS and ES. The results revealed that MC is the most significant factor that influences ES among women-owned SMEs. Their specific personality dispositions are the second important contributor that help women achieve entrepreneurial success. These are followed by AFR and GS, which are the 3^rd^ and 4^th^ important factors when it comes to achieving ES. The results also revealed that MC plays an intermediate role in the link between PT and ES, highlighting that without specific personality dispositions, these women entrepreneurs cannot develop MC. Therefore, PT is important to keep higher levels of MC among the women-owned SMEs and direct them towards achieving ES. Such factors also assist in boosting women’s entrepreneurial sustainability in developing nations.

### 7.1. Research implications

On the basis of the study findings, we present practical implications that can assist multiple government institutions, such as departments related to women empowerment, SMEs, financial institutions, and other prospective women entrepreneurs, for the improvement and growth of women-owned SMEs. These findings guide policymakers and facilitate women entrepreneurs to ensure that women can play a significant role in their country’s sustainable economic development. These results can also guide government authorities in empowering women and improving their motivation and spirit to overcome the challenges faced in running businesses through trainings on managing financial matters, and moral encouragement. The results suggest that the government should devote more resources to educating and empowering women business owners so that they are better equipped to take on new challenges in the business world.

Our study confirms that the AFR is an important factor when establishing the businesses. Therefore, banks and financial institutions must revise their policies on the appropriate allocation of financial resources, reduce credit procedures, and charge minimum interest rates, particularly toward women-owned SMEs. Doing so can improve the success rate of such SMEs in Pakistan. In order to become an entrepreneur, women should find their strengths and opportunities in the external environment. Here, motivation plays an important role in recognizing their strengths, leading them to become a successful entrepreneur. Having a positive mindset, attitude, knowledge, and passion can stimulate and cultivate the emotional attachment of women entrepreneurs to their goals, thereby maintaining their motivation and commitment to succeed. Moreover, not everybody is born with the wisdom to transform the world. Every entrepreneur needs to overcome several problems to succeed in business; whether it is not getting sufficient resources, proving that the opponent is wrong, or facing the contest head-on, it is not easy to be an entrepreneur. This is why women entrepreneurs need to be passionate, and flexible to adopt challenges and have a broad vision to sense the opportunities which lead towards ES. Furthermore, potential and existing women entrepreneurs should start and expand their business operations by obtaining support from the government, NGOs, and other financial institutions to reduce these challenges.

### 7.2. Limitations and future recommendation

Apart from the significant contributions of this study, its limitations may reduce the generalization due to the sample size, geographical spread, and time constraints of the target subjects. Therefore, a longitudinal study should be conducted to investigate the improvement of community policies for women entrepreneurs and further economic changes in Pakistan. In addition, the personality traits were measured using the six items scale. Future studies are strongly encouraged to use big five personality traits (Hachana et al., 2018) and find out the most influential personality trait for ES. Future researchers can also study socio-cultural factors like married and not-married fields (Chowdhury, 2012), norms, values, and other cultural boundaries, which influence women’s decision to become successful entrepreneurs, particularly in developing countries. Pakistan is a country with a majority of its population living in rural areas. Therefore, future researchers can differentiate the performance of women entrepreneurs based on rural and urban areas (Muhammad and Ximei, 2022). Such a comparison is likely to provide useful information to governments and policymakers for addressing the main obstacles faced by women entrepreneurs.

## Data availability statement

The original contributions presented in the study are included in the article/supplementary material, further inquiries can be directed to the corresponding author.

## Ethics statement

Ethical review and approval was not required for the study on human participants in accordance with the local legislation and institutional requirements. The patients/participants provided their written informed consent to participate in this study.

## Author contributions

JF: resources, project administration, supervision, and funding acquisition. ZA: conceptualization, data collection, and writing the draft. WZ: formal analysis, methodology, writing, review, and editing. All authors contributed to the article and approved the submitted version.

## Funding

This work was supported from the Heilongjiang Province Philosophy and Social Science Research and Planning Project (Project No. 22GLC279) and from the 2022 Young Doctor Research Launch Fund project of Harbin University (HUDF2022113). This support is gratefully acknowledged.

## Conflict of interest

The author declares that the research was conducted in the absence of any commercial or financial relationships that could be construed as a potential conflict of interest.

## Publisher’s note

All claims expressed in this article are solely those of the authors and do not necessarily represent those of their affiliated organizations, or those of the publisher, the editors and the reviewers. Any product that may be evaluated in this article, or claim that may be made by its manufacturer, is not guaranteed or endorsed by the publisher.
